# Roles of Dental Mesenchymal Stem Cells in the Management of Immature Necrotic Permanent Teeth

**DOI:** 10.3389/fcell.2021.666186

**Published:** 2021-05-19

**Authors:** Dixin Cui, Sihan Yu, Xin Zhou, Ying Liu, Lu Gan, Yue Pan, Liwei Zheng, Mian Wan

**Affiliations:** ^1^State Key Laboratory of Oral Diseases, National Clinical Research Center for Oral Diseases, Department of Pediatric Dentistry, West China Hospital of Stomatology, Sichuan University, Chengdu, China; ^2^State Key Laboratory of Oral Diseases, National Clinical Research Center for Oral Diseases, Department of Cariology and Endodontics, West China Hospital of Stomatology, Sichuan University, Chengdu, China

**Keywords:** dental mesenchymal stem cells, regenerative endodontics, pulp-dentin regeneration, immature permanent teeth, cell transplantation

## Abstract

Dental caries and trauma always lead to pulp necrosis and subsequent root development arrest of young permanent teeth. The traditional treatment, apexification, with the absence of further root formation, results in abnormal root morphology and compromises long-term prognosis. Regeneration endodontics procedures (REPs) have been developed and considered as an alternative strategy for management of immature permanent teeth with pulpal necrosis, including cell-free and cell-based REPs. Cell-free REPs, including revascularization and cell homing with molecules recruiting endogenous mesenchymal stem cells (MSCs), have been widely applied in clinical treatment, showing optimistic periapical lesion healing and continued root development. However, the regenerated pulp–dentin complex is still absent in these cases. Dental MSCs, as one of the essentials of tissue engineering, are vital seed cells in regenerative medicine. Dental MSC–based REPs have presented promising potential with pulp–dentin regeneration in large animal studies and clinical trials via cell transplantation. In the present review, we summarize current understanding of the biological basis of clinical treatments for immature necrotic permanent teeth and the roles of dental MSCs during this process and update the progress of MSC-based REPs in the administration of immature necrotic permanent teeth.

## Introduction

Immature permanent teeth are prone to pulpal necrosis due to caries, trauma, or developmental malformation. These cases always lead to arrest of root formation, accompanied by thin root dentinal walls and open apices, which has been a challenge in endodontics (Shabahang, 2012). With apexification, the traditional treatment, either calcium hydroxide or mineral trioxide aggregate (MTA) is applied to achieve apical sealing (Andreasen and Bakland, 2011; Nicoloso et al., 2017). Apexification has been reported to resolve apical periodontitis with a success rate of 74–100% (Al Ansary et al., 2009). However, absence of further root formation with apexification still results in abnormal root morphology, such as thin dentinal walls with an increased risk of root fracture, consequently compromising long-term prognosis (Rafter, 2004).

Regeneration endodontics procedures (REPs) have been developed and considered as an alternative strategy for treatment of immature permanent teeth with pulp necrosis (Murray et al., 2006). The notion of tissue regeneration in the root canal was first proposed in the 1960s (Nygaard-Ostby, 1961). Banchs and Trope (2004) introduced a case report describing an alternative treatment for the management of necrotic immature permanent teeth called revascularization, in which a blood clot was induced inside the root canal after control of inflammation. Later, autologous platelet-rich plasma (PRP) and platelet-rich fibrin (PRF) took the place of the blood clot as alternative scaffolds because of their potential to induce tissue regeneration (Lovelace et al., 2010). A standard protocol for clinical REPs was proposed by the American Association of Endodontists [AAE], 2016b) in 2016 These REPs without exogenous cells, including revascularization and cell homing, have been successful in resolving apical periodontitis and arrest of root formation (Iwaya et al., 2001; Torabinejad and Turman, 2010; Shimizu et al., 2013). However, histological studies show that the pulp–dentin complex is absent in these cases although some of them have shown vital pulp (Shimizu et al., 2013; Ulusoy et al., 2019). Desired REPs are supposed to eliminate apical periodontitis; increase root length, dentinal wall thickness, and apical closure; and restore homeostatic function of the pulp–dentin complex, including inherent immunity, tertiary dentin formation with stimulus, and pulp sensibility. In particular, the reinstitution of pulp–dentin structure functions prolongs the life of the tooth. Hence, scientists and endodontists are keen to develop a novel regenerative strategy to achieve pulp vitality and organized pulp–dentin structure with homeostatic functions.

Three major elements have been recommended by Diogenes for further studies of pulp–dentin regeneration, including (i) reliable cell resources responsible for formation of root dentin, pulp tissue, and supporting tissue; (ii) an applicable scaffold to facilitate cellular proliferation and differentiation; and (iii) signaling molecules to motivate and direct tissue development, maturation, and neovascularization (Diogenes et al., 2016). Mesenchymal stem cells (MSCs) responsible for pulp–dentin regeneration might be indispensable for ideal REPs. Several preclinical studies reveal the regenerative potential of pulp–dentin tissue via cultured cell transplantation (Nakashima et al., 2017; El Ashiry et al., 2018; Xuan et al., 2018). With its accessibility and unique potential in dental tissue regeneration, including the pulp–dentin complex, dental MSCs play a decisive role of seed cells in REPs. In this context, the applications of dental pulp stem cells (DPSCs), stem cells from human exfoliated deciduous teeth (SHED), stem cells from apical papilla (SCAP), periodontal ligament stem cells (PDLSCs), and dental follicle stem cells (DFSCs) have been explored. In the latest clinical study (Xuan et al., 2018), implantation of autologous SHED aggregates generated pulp–dentin complex in immature necrotic permanent incisors of pediatric patients, including functional dental pulp tissue regeneration with vasculature, innervation, and the lining odontoblast layer. The regenerated dental pulp tissue promotes root elongation and apical foramen closure. Therefore, dental MSCs exert therapeutic applications and are of great importance in treating immature necrotic permanent teeth.

In this review, we briefly summarize the current understanding of the biological basis of clinical treatments for immature permanent teeth with pulpal necrosis and the roles of dental MSCs during this process and update the progress of MSC-based REPs in the treatment of immature necrotic permanent teeth.

## Biological Basis for REPs

**FIGURE 1:**
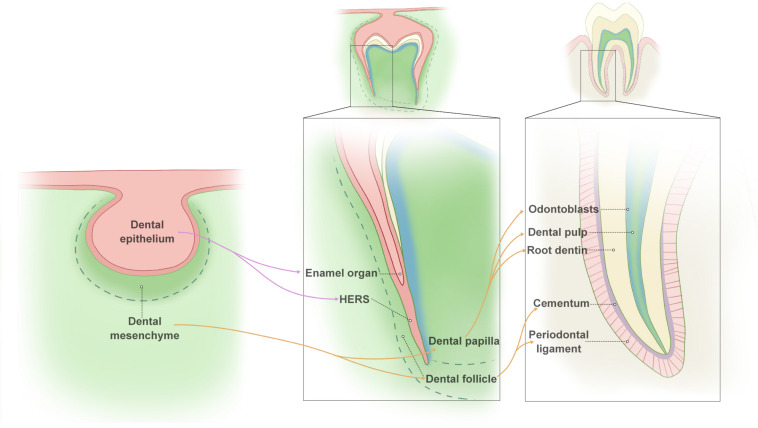
Summary of root formation.

Root development relies on temporospatial reciprocal action between dental epithelium (Hertwig’s epithelial root sheath, HERS) and mesenchyme from the cranial neural crest (dental papilla and follicle) (Thesleff and Sharpe, 1997). When the tooth crown is formed, HERS is formed by the inner and outer enamel epithelium of the enamel organ, which lies between the dental papilla and follicle. Then, HERS extends apically with the dental papilla and follicle and eventually regulates root formation. The inner epithelial cells of HERS induce MSCs at the periphery of the pulp to form odontoblasts, which produce the root dentin (Huang et al., 2009). As SHED fragments, the dental follicle penetrates into the epithelial fenestrations, contacts the root dentin, and differentiates into cementoblasts, which form the cementum covering the root dentin (Zeichner-David et al., 2003; Sonoyama et al., 2007b; Huang et al., 2009). The dental follicle is also responsible for the formation of periodontal ligament and fiber bundles. Hence, HERS plays a vital role in the interaction between the dental epithelial and dental mesenchymal compartment during root formation (Figure 1).

Arrested root formation in immature necrotic permanent teeth is always related to severe HERS damage due to dental trauma (Andreasen et al., 1988). Inflammatory cytokines and chemokines induced by severe and chronic inflammation impair the stem cells during tissue repair (Cooper et al., 2014). Additionally, function of SCAP could be disrupted by proinflammatory cytokines (Johnson, 1997; Liu et al., 2016; Wang et al., 2017). Hence, root formation is halted in immature permanent teeth with pulpal necrosis. Once inflammation is controlled, proinflammatory cytokine and chemokines are reduced, which leads to resumption of the regulatory effect of HERS and, consequently, induces the continued formation of the incomplete root (Cooper et al., 2010; Diogenes and Hargeraves, 2017). MSCs are vulnerable to the inflammatory microenvironment, and their immunomodulatory capacities can vary unexpectedly with the exposure to different inflammatory conditions (Noronha et al., 2019). It is demonstrated that the TNFα/TNFR2 signaling pathway is involved in regulating the immunomodulatory properties of MSCs (Beldi et al., 2020a, b). The TNFα–TNFR2 axis mediates MSCs’ anti-inflammatory effects and cell survival, indicated by the inhibition of T cell proliferation, the production of proinflammatory cytokines, and the inductive activation of regulatory T cells. The presence of the TNFR2 molecule is also involved in the regulatory effect of MSCs, such as the colony-forming unit, proliferation, and MSC-specific surface markers. TNFR2 is expressed predominantly in endothelial progenitor cells. The TNFα/TNFR2 signaling pathway is also critical in the regulation of endothelial progenitor cell immunosuppression and the angiogenic effect to form new immunosuppressive vessels (Naserian et al., 2020). Whether the inflammatory environment caused by pulpal necrosis/apical periodontitis plays protective and essential roles in the biological functions of SCAPs needs further study. A previous study suggests the long-term viability of apical papilla under prolonged root canal infection and apical periodontitis (Diogenes and Hargeraves, 2017). On the contrary, MSCs responsible for the pulp–dentin complex in the root canal rarely survive during chronic endodontic infection, which explains the absence of pulp–dentin regeneration with revascularization or cell homing.

## Dental Mesenchymal Stem Cells

Human MSCs are multipotent cells from various tissues, such as skeletal muscle, adipose tissue, placenta, bone, and dental tissue (Pittenger et al., 1999). Based on minimal criteria proposed by the International Society for Cellular Therapy (ISCT), MSCs are plastic-adherent; possess multilineage differentiation potential *in vitro*; express at least CD105, CD73, and CD90; and negatively express CD11b, CD14, CD19, CD34, CD45, CD79α, and HLA-DR cell surface markers (Dominici et al., 2006; Han et al., 2019). According to MSC minimal criteria, dental MSCs derived from dental tissues, including impacted teeth and their supporting tissues, have been identified and characterized with typical MSC properties (Sharpe, 2016; Table 1). In addition to easy access, dental MSCs are genomically stable after multiple passages *in vitro*. Despite their multilineage differentiation capacity, dental MSCs are distinct from other MSCs because of the unique potential in dental tissue regeneration and have aroused much interest in regenerative medicine, especially the applications of REPs (Huang et al., 2009a). To date, dentin, dental pulp, or even pulp–dentin complex-like structure regenerations with the application of dental MSCs have been widely investigated, including DPSCs, SHED, SCAP, PDLSCs, and DFSCs.

**TABLE 1:** Comparison of root formation-related dental MSCs in immature permanent teeth.

	**Source**	**Surface marker positive**	**Surface marker negative**	**Multi-lineage differentiation potential**	**Immunomodulatory properties**
DPSCs	Dental pulp tissue of permanent teeth	CD9, CD10, CD13, CD29, CD44, CD59, CD73, CD90, CD105, CD106, CD146, CD166, STRO-1, NANOG, SOX2, OCT4, TRA1-60, TRA-1-80-1, and Nestin	CD14, CD19, CD24, CD31, CD34, CD45, and CD117	odontoblasts, osteoblasts, chondrocytes, adipocytes, neurons, cardiomyocyte, and hepatocytes	Immunosuppressive properties increased HGF, TGF-β, PGE-2, IL-6, and IDO; decreased IL-4 and IFN-γ; suppressed proliferation of T cells and PBMCs; increased number of regulatory T cells
SHED	Dental pulp tissue of exfoliated deciduous teeth	CD13, CD29, CD44, CD73, CD90, CD105, CD146, STRO-1, NANOG, adn Nestin	CD14, CD15, CD19, CD34, and CD45	odontoblasts, osteocytes, chondrocytes, adipocytes, neurons, and hepatocytes	Immunosuppressive properties increased IL-10; decreased IL-4 and IFN-γ; inhibited Th17 cell differentiation; increased number of regulatory T cells
SCAP	Apical papilla	CD13, CD24, CD29, CD44, CD49, CD51, CD56, CD61, CD73, CD90, CD106, CD146, CD166, STRO-1, NANOG, and Nestin	CD14, CD18, CD34, and CD45	odontoblasts, osteocytes, adipocytes, neurons, and hepatocytes	Low immunogenicity inhibited proliferation of T cells
PDLSCs	Periodontal ligament	CD9, CD10, CD13, CD29, CD44, CD59, CD73, CD90, CD105 CD106, CD146, CD166, and STRO-1	CD14, CD19, CD34, CD45, and HLA-DR	cementoblasts, osteoblasts, chondrocytes, adipocytes, and neurons	Immunosuppressive properties expressing TLR2 and TLR4; released HGF, TGF-β, and IDO; suppressed proliferation of PBMCs
DFSCs	Dental follicle	CD9, CD10, CD13, CD29, CD44, CD59, CD73, CD90, CD105, CD106, CD146, CD166, STRO-1, NANOG, SOX2, OCT4, and Nestin	CD31, CD34, CD45, and CD133	odontoblasts, cementoblasts, osteoblasts, chondrocytes, adipocytes, neurons, and cardiomyocytes	Immunosuppressive properties expressing TLR2, TLR3, and TLR4; increased IL-6, TGF-β, and IDO-1; decreased IFN-γ, IL-4, and IL-8; suppressed proliferation and apoptosis of PBMCs; increased number of regulatory T cells

*DPSCs, dental pulp stem cells; SHED, stem cells of human exfoliated deciduous teeth; SCAP, stem cells from apical papilla; PDLSCs, periodontal ligament stem cells; DFSCs, dental follicle stem cells; HFG, hepatocyte growth factor; IDO, indole amine 2,3-dioxygenase; IFN, interferon; IL, interleukin; PGE2, prostaglandin E2; TGF-β, transforming growth factor beta; Th17, T-helper 17; TLR, Toll-like receptor; PBMCs, peripheral blood mononuclear cells.*

### DPSCs

Dental pulp tissue, formed by neural crest–derived dental papilla, is the soft tissue surrounded by the dentin. Responding to external stimuli, odontoblasts form the tertiary dentin. These odontoblasts are supposed to be derived from the progenitor cell populations within dental pulp. DPSCs, first isolated from adult third molar pulp tissues by Gronthos et al., possess definitive MSC characteristics, self-renewal capacity, and multilineage differentiation potential (Gronthos et al., 2000). DPSCs can differentiate into osteoblast-like cells with specific markers, forming new bone *in vivo* (Mortada and Mortada, 2018). A series of case reports indicate the potential application of DPSCs in treating intraosseous defects. In these patients with such defects caused by periodontitis, minimally invasive flap and collagen sponge integrated with autologous/allogeneic DPSCs have been applied. Results showed decreased probing depth reduction, achievement of clinical attachment, and formation of new bone with rare adverse effects, indicating the periodontal tissue regeneration potential of DPSCs (Aimetti et al., 2018; Ferrarotti et al., 2018; Hernández-Monjaraz et al., 2018). DPSCs are known to differentiate into odontoblasts that are indispensable for dentinogenesis. Dentin-like tissue is formed *in vivo* with DPSCs and hydroxyapatite/tricalcium phosphate (HA/TCP) scaffold, presenting a lining odontoblast-like cell layer of a specific odontoblastic-related marker expression, dentin sialophosphoprotein (DSPP) (Anitua et al., 2017; da Silva et al., 2019). Compared with human bone marrow MSCs, DPSCs exhibit notable neurogenic potential due to their origin of the neural crest and could differentiate into neurons upon specific differentiation induction (Pagella et al., 2019). The neurogenic potential was also confirmed with higher expression levels of neurotrophins when DPSCs were cocultured with trigeminal neurons (Jung et al., 2016; Kawase-Koga et al., 2019). DPSCs also display angiogenic potential for differentiation potential of endothelial cells and the formation of blood vessels after *in vivo* transplantation with HA scaffold (Jeong et al., 2020). A clinical study shows that implanted DPSCs achieve pulp-like tissue regeneration with vasculature and innervation in the root canal of traumatized incisors (Nakashima et al., 2017). The potential application of DPSCs in dental pulp tissue regeneration has also been indicated by another case report. The affected mature permanent tooth with symptomatic irreversible pulpitis shows a positive response in pulp vitality test, following the administration of autologous DPSCs and leukocyte PRF in the root canal of the affected tooth (Meza et al., 2018). The promising neurogenic, angiogenic, and odontoblastic differentiation potential makes DPSCs a major contributor to dentin regeneration and even whole pulp regeneration.

### SHED

SHED were collected from children’s exfoliated deciduous teeth with a similar methodology as that for DPSCs (Miura et al., 2003). SHED possess multilineage differentiation potential and can differentiate into various cell types, such as odontoblasts, adipocytes, and neurons (Miura et al., 2003). However, SHED show capacities of higher proliferation, more cell population doublings, and remarkable osteoinduction compared with DPSCs based on developmental differences between deciduous and permanent teeth. Regenerated new bone with larger osteoids and more collagen fibers by SHED with a polylactic-coglycolic acid membrane suggests that SHED exhibit outstanding potential for bone regeneration compared with DPSCs and bone marrow MSCs (Miura et al., 2003; Kunimatsu et al., 2018). As for the potential of neural regeneration, SHED show more intensive expression of neural differentiation markers than DPSCs under neural induction culture, such as β-III-tubulin and nestin (Wang et al., 2009) and can also promote neural functional recovery (Nicola et al., 2018). The odontoblastic differentiation capacity of SHED has been confirmed by *in vivo* transplantation that the composites of SHED and HA/TCP form a dentin-like structure containing DPSS-positive odontoblasts (Miura et al., 2003). SHED are also capable of forming functional dental pulp tissue, containing odontoblasts to regenerate tubular dentin in full-length root canals combined with collagen type I (Cordeiro et al., 2008). The abovementioned odontoblastic differentiation capacity renders SHED a promising cell source for dentin or pulp regeneration; and whole dental pulp regeneration has been achieved by SHED (Xuan et al., 2018).

### SCAP

In the process of tooth development, dental papilla forms dental pulp and migrates apically (Sonoyama et al., 2007b). Several clinical case reports show that root formation continues in some necrotic immature permanent teeth, indicating that MSCs in apical papilla contribute to root development. SCAP are obtained from apical papilla of immature tooth roots and exhibit MSC properties, including expression of MSC surface markers and differentiation potential to a wide variety of cell types (Sonoyama et al., 2006, 2007a). SCAP possess neural differentiation potential similar to DPSCs and SHED, partially attributed to their common origin from the neural crest, and could be an alternative future therapy for spinal cord injury (De Berdt et al., 2015). Interestingly, SCAP have higher proliferation and greater odontoblastic differentiation potential than DPSCs, suggesting their potential applications for dentin regeneration (Sonoyama et al., 2006). *In vivo* studies show that SCAP are able to differentiate into odontoblast-like cells and generate dentin-like tissue with DSP expression (Sonoyama et al., 2006, 2007a). The dentin regeneration capacity of SCAP via cell homing strategy is enhanced by their greater migration ability following a scratch assay. SCAP can also form ectopic vascularized pulp-like tissue with DSPP and dentin matrix protein 1 (DMP1)- positive odontoblasts in mouse molars without exogenous growth factor application (Pelissari et al., 2018). Owing to their critical role in root development, SCAP are supposed to make a major contribution to root regeneration. After transplantation of SCAP and PDLSCs into a minipig model with a lower incisor extracted, a functional bioroot with root/periodontal–like complex was formed. Mineralized root-like tissue is able to support a porcelain crown and perform normal tooth function (Sonoyama et al., 2006).

### PDLSCs

A population of MSCs exists in the periodontal ligament (PDL), and it is responsible for periodontal tissue homeostasis and regeneration (McCulloch and Melcher, 1983; Seo et al., 2004). These cells were first isolated from the PDL of third molars and named PDLSCs. The cementogenic/osteogenic differentiation potential is indicated by the formation of mineralization nodules with the expression of bone-specific markers after *in vivo* transplantation (Seo et al., 2004). The cementogenic/osteogenic differentiation potential and PDL tissue regeneration potential of PDLSCs are shown in a rat model of periodontal lesions, confirmed by newly formed cementum/PDL-like structures at the lesion area, such as Sharpey’s fiber-like tissue (Seo et al., 2004; Iwata et al., 2010). A recent preclinical study using a novel cell transfer technology demonstrates the potential of PDLSCs in periodontal regeneration. In a rat model of surgical periodontal defects, the transplantation of PDLSC-amniotic membrane composite enhanced the periodontal defect recovery, manifested as newly formed PDL, bone, and cementum at surgically defective sites (Iwasaki et al., 2019).

### DFSCs

Dental follicle contributes to alveolar bone formation during tooth development, and contains an MSC population to form supporting tissues, named DFSCs. DFSCs were separated from the dental follicle of developing teeth (Morsczeck et al., 2004; Han et al., 2009; Zhou et al., 2019). Compared with DPSCs, SHED, and PDLSCs, DFSCs show a higher proliferation and colony-forming capacity, indicating their application potential in regenerative medicine (Tian et al., 2015; Yildirim et al., 2016). DFSCs also exhibit superior osteogenic properties compared with DPSCs and SHED as shown by the higher expression levels of osteogenic genes (Yildirim et al., 2016). Under the administration of differentiation induction culture medium, DFSCs form osteoblasts and produce mineralized nodules with osteogenic differentiation marker expression, bone sialoprotein, and osteocalcin (Morsczeck et al., 2004; Han et al., 2009). DFSCs are capable of periodontal differentiation, indicated by the formation of PDL-like tissues or mineralized structures with bone- or cementum-like tissues (Morsczeck et al., 2004; Han et al., 2009). DFSCs generate complex tissues similar to cementum-PDL complex *in vivo*, in which PDL-like collagen fibers are inserted into newly formed cementum-like tissue (Han et al., 2009). The potential of odontoblastic differentiation has also been suggested in DFSCs because they have been shown to express higher level of DSPP compared with PDLSCs. The formation of dentin, including dentin, predentin, and calcospherites, is observed with treated dentin matrix induction (Trubiani et al., 2019). All these findings suggest DFSCs as promising seed cells for both dentin and root regeneration.

## Cell-Free REPs

### Roles of Dental MSCs in Cell-Free REPs

The first attempt at dental pulp tissue regeneration was proposed by Nygaard-Otsby et al. (Nygaard-Ostby, 1961; Nygaard-Ostby and Hjortdal, 1971). Over-instrumentation was applied to introduce blood from the periapical tissues into the root canal, followed by tissue growth. Later, Banchs and Trope (2004) proposed a protocol termed revascularization based on the experiments of Kling et al. (1986) on implanted teeth, Hoshino et al. (1996) on root canal disinfection, and (Nygaard-Ostby and Hjortdal, 1971) on blood clots in the canal space.

The standard REP protocol proposed by the American Association of Endodontists [AAE] (2016b; 2018) involves a multistep procedure. The first visit focuses on infection control of the affected tooth with the administration of a proper access cavity, canal irrigation, and disinfection. The common root canal dressing is calcium hydroxide or triple antibiotic paste (TAP), which is a mixture of ciprofloxacin, metronidazole, and minocycline. The second appointment aims to form the suitable scaffold formation for fresh tissue ingrowth and permanent coronal restoration following the absence of clinical signs and symptoms. During this appointment, the root canal is thoroughly irrigated with ethylenediaminetetraacetic acid to release the growth factor from the dentin. Apical bleeding is then stimulated by gentle irritation with a precurved K-file at 2 mm past the apical foramen to form a blood clot in the root canal. Finally, capping material, usually MTA, is placed over the blood clot, followed by the permanent coronal seal to prevent bacterial reinfection. At the follow-up, eliminating clinical signs and symptoms and healing periapical lesion are considered as primary goal of REPs. It is desirable, but not essential, that REPs increase the thickness of the root wall and/or length of the roots, which is the secondary goal. Some cases report that the teeth showed a positive response to pulp vitality testing, suggesting organized pulp tissue in the root canal, which achieves the tertiary goal.

**FIGURE 2:**
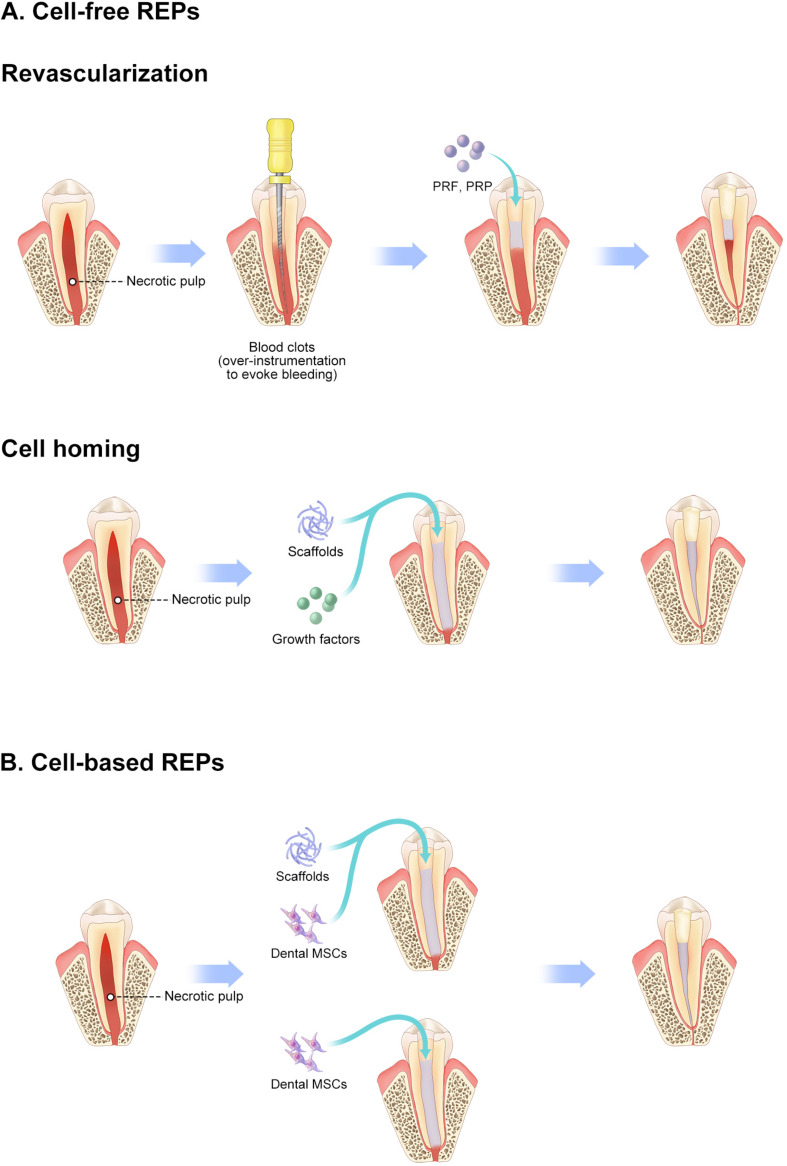
Schematic diagram of REPs. **(A)** Cell-free REPs. **(B)** Cell-based REPs. REPs, regeneration endodontics procedures.

As an amelioration to revascularization with blood clots, the cell homing strategy has been proposed to regenerate dental tissue via a cell-free strategy in which molecules encourage recruitment of the patient’s endogenous MSCs to the root-canal space (He et al., 2016; Yin et al., 2017). Several endodontists believe that cell homing is conducive to achieving a more effective strategy of pulp–dentin regeneration than simple revascularization without exogenous cell transplantation (Table 2). Several molecules, including basic fibroblast growth factors, vascular endothelial growth factors, platelet-derived growth factors, nerve growth factors, and bone morphogenetic protein 7, have been applied as homing factors, showing promising outcomes in preclinical studies (Kim et al., 2010). These REPs without exogenous cell transplantation, including revascularization and cell homing, are considered cell-free REPs. Survival rates of cell-free REPs are reported close to 100% in some studies. Therefore, these studies suggest that cell-free REPs have an obvious therapeutic effect on necrotic immature teeth (Figure 2A).

**TABLE 2:** Current preclinical and clinical studies of cell-free REPs.

**Study/year**	**Sample size (teeth)**	**Animal model**	**Intracanal medication**	**Scaffold**	**Capping material**	**Observation period**	**Results or outcomes**
**Preclinical studies**							
da Silva et al., 2009	40	Dogs	TAP	Empty scaffold	MTA	90 days	Hard tissue barrier, and increase of apical periodontal ligament thickness
Yamauchi et al., 2010	64	Dogs	TAP	Cross-linked collagen scaffold + blood clot, blood clot	MTA	3.5 months	Periapical healing and root wall thickening
Tawfik et al., 2013	108	Dogs	TAP	bFGF injectable scaffold + blood clot, blood clot	MTA	3 months	Negative results in this study: no change of root length and root thickness.
Khademi et al., 2014	36	Dogs	TAP	Blood clot	MTA	3-6 months	periapical healing, apical closure, and dentinal walls thickening
Yoo et al., 2014	30	Dogs	TAP	a collagen scaffold sponge (soaked with conditioned media from mouse preameloblasts) + blood clot	MTA	12 weeks	Continuous growth of root dentin, and hard tissue formation
Zhang et al., 2014	27	Dogs	TAP	PRP, blood blot	MTA	3 months	Root canal walls thickening, and apical closure
Londero Cde et al., 2015	20	Dogs	TAP	Gelatin-based scaffold (Gelfoam) + blood clot, blood clot	MTA	7 months	Increase in root length
Rodríguez-Benítez et al., 2015	40	Dogs	modified triple-antibiotics paste (mTAP)	PRP, blood blot	MTA	6 months	Root dentinal walls thickening, hard tissue deposition on dentinal walls, and apical closure
Saoud et al., 2015	17	Dogs	TAP	Blood clot	MTA	3 months	Not reported about root development and apical closure; but significant dentinal walls thickening, and periapical healing
Torabinejad et al., 2015	24	Dogs	TAP	Blood clot/Gelfoam, PRP	MTA	3 months	Apical narrowing, and hard tissue deposition in the apical third of the root
Altaii et al., 2017	4	Sheep	TAP	Blood clot	MTA	6 months	Significant increases in root length, root wall thickness and narrowing of root canal apical diameter

**Study/year**	**Sample size (teeth)**	**Age of patients (mean ± SD)**	**Intracanal medication**	**Scaffold**	**Capping material**	**Observation period (mean ± SD)**	**Results or outcomes**

**Clinical studies**							
Reynolds et al., 2008	2	11 years old	TAP	Blood clot	MTA	18 months	Significant root development with maturation of the dentin
Bose et al., 2009	88	-	TAP, Ca(OH)2, and formocresol	Blood clot	MTA	6 months-36 months	Continued root development: increased percentage of root length and dentinal wall thickness
Ding et al., 2009	12	8–11 years old	TAP	Blood clot	MTA	15 months	3 teeth of 12 exhibit complete root development with closed apex and positive response to electric pulp testing
Petrino et al., 2009	6	6, 11, and 13 years old	TAP	Blood clot	MTA	6-12 months	3 of 6 teeth showed continued root development, and 2 teeth displayed positive response to vitality testing
Iwaya et al., 2010	1	7 years old	Ca(OH)_2_ paste (Vitapex)	Empty scaffold	Gutta-percha	30 months	Continued root development, root apex closure, and root canal thickness increase
Thomson and Kahler, 2010	1	12 years old	TAP	Blood clot	MTA	18 months	Continued root maturation and apical closure
Torabinejad and Turman, 2010	1	11 years old	TAP	PRP	MTA	5.5 months	Periapical lesion resolution, further root development, and continued apical closure of the root apex
Chen et al., 2011	20	8-13 years old	Ca(OH)_2_	Blood clot	MTA	6-26 months	periapical wound healing, and Increased thickening of root canal walls; 15 of 20 teeth continued root development; 4 of 20 teeth exhibited severe hard tissue calcification in pulp canal; 2 of 20 teeth formed a hard tissue barrier in root canal space
Nosrat et al., 2011	2	8, and 9 years old	TAP	Blood clot	Calcium enriched mixture (CEM)	15-18 months	Periapical radiolucent lesions healing, and continued roots development
Jeeruphan et al., 2012	20	8-24 years old	TAP	Blood clot	MTA	21.15 ± 11.70 months	Increased percentage of root width and root length
Kim et al., 2012	3	10 and 12 years old	TAP	Blood clot	MTA	24, 42, and 48 months	Periapical radiolucency disappeared, and root wall thickness increased
Martin et al., 2012	1	9 years old	TAP	PRP + Blood clot	MTA	2 years and 1 months	Resolution of apical periodontitis; hard tissue of obliteration in distal canal, reduction in size of mesial canal space
Jadhav et al., 2013	6	10, 13, and 23 years old	TAP	PRP + blood clot, blood clot	Resin modified glass ionomer cement	12 months	Periapical healing, apical closure, and dentinal wall thickening
Kahler et al., 2013	16	7-12 years old	TAP	Blood clot	MTA	18 months	Patterns of continued root maturogenesis were variable: 90.3% resolution of the periapical radiolucency, 47.2% incomplete apical closure, 19.4% complete apical closure, 2.7% to 25.3% change of root length, and 1.9% to 72.6% change of root dentin thickness
Nagy et al., 2013	36	9-13 years old	TAP	FGF + blood clot, blood clot	MTA	18 months – 3 years	Periapical healing, increase in root length and width, and a decrease in apical diameter
Shimizu et al., 2013	1	9 years old	Ca(OH)_2_	Blood clot	MTA	26 months	Resolution of periapical lesion, continued root development, thickening of the canal walls
Sönmez et al., 2013	3	9 years old	TAP	Blood clot	MTA	24 months	Continued thickening of the dentinal walls with apical closure; complete resolution of periapical radiolucencies
Alobaid et al., 2014	31	6-16 years old	TAP, BAP, Ca(OH)_2_	Blood clot	MTA	14.5 ± 8.5 months	Apical closure and hard tissue barrier; but a greater incidence of adverse events in revascularization group
Bezgin et al., 2014	22	7–13 years old	TAP	PRP, blood clot	MTA	18 months	Complete apical closure, periapical tissue pathology resolution
Nagata et al., 2014	23	7-17 years old	TAP, Ca(OH)_2_, and chlorhexidine	Blood clot	MTA	9-19 months	Periapical repair, apical closure, root length increase, dentinal walls thickening; but crown discoloration in teeth of TAP group
Saoud et al., 2014	20	11.3 ± 1.9 years old	TAP	Blood clot	MTA	1 year	Increase in radiographic root width and length and decrease in apical diameter
Narang et al., 2015	20	Below 20 years old	TAP	RPF + blood clot, PRP + collagen, blood clot	Resin-modified glass ionomer cement	6-18 months	PRF shows significant periapical healing, apical closure, root lengthening, and dentinal wall thickening in revascularization treatment
Nosrat et al., 2015	2	9, 10 years old	TAP	Blood clot	MTA	4 months	Progression of root development and maturation of the roots
Timmerman and Parashos, 2016	1	16 years old	Ca(OH)_2_	Blood clot	MTA	3 years	Complete periapical healing, thickening of the dentinal root walls, and completed apex formation
Austah et al., 2018	2	8, and 10 years old	BAP, Ca(OH)_2_	Blood clot + CollaPlug	MTA	43-54 months	Complete healing of periapical tissues, continued root development, root length increase, and dentin thickness increase
Ajram et al., 2019	1	7 years old	Ca(OH)_2_	Blood clot	Micro Mega- MTA (MM-MTA)	2 years	Complete apical healing, continued root growth, and apical closure
Rizk et al., 2019	26	8-14 years old	TAP	PRP, blood clot	MTA	12 months	Significant increase in root length, root width, and decrease in apical diameter of PRP-treated teeth compared with blood clot group; but higher amount of crown discoloration in blood clot-treated teeth
Alasqah et al., 2020	1	8 years old	Ca(OH)_2_, TAP	Blood clot	MTA	24 months	Periapical healing with increased root thickness and length, and complete apical closure

*BAP, bi-antibiotics paste; bFGF, basic fibroblast growth factor; Ca(OH)2, calcium hydroxide; MTA, mineral trioxide aggregate; PRF, platelet-rich fibrin; PRP, platelet-rich plasma; TAP, triple-antibiotics paste.*

Stem cells, homing to the injury site, have an essential role in wound healing (Rustad and Gurtner, 2011). The cells in the sites of injury and inflammation release chemokines, stem cell factors, and growth factors, which motivate the cell homing (Eramo et al., 2018). CXCR4^+^ SCAP are demonstrated to be chemoattracted by stromal derived factor 1, a chemokine, and migrate into a scaffold made of collagen gel (Liu et al., 2015). In cell-free REPs, stem cells from the periapical tissues get into the root canal space in various ways, mainly by periapical bleeding and molecules in the scaffolds. It is demonstrated that a large number of MSCs with expression of CD105, CD73, and STRO1 were induced into the empty root canal by importing periapical bleeding (Lovelace et al., 2010). These cells were supposed to be MSCs from the adjacent apical papilla rather than systemic circulation although no direct evidence is shown in that study. Additionally, histological and immunohistochemical analysis presented the formation of cementum- and bone-like structures in necrotic immature permanent teeth with cell-free REPs. It suggests that stem/progenitor cells in periapical tissue, responsible for production of cementum and bone, also entered the root canal and participated in the formation of mineralized tissue during continued root formation (Martin et al., 2012; Shimizu et al., 2012, 2013; Torabinejad and Faras, 2012; Becerra et al., 2013; Nosrat et al., 2015). Therefore, undifferentiated MSCs originated from apical papilla, and periapical tissues are considered to be major cell sources for continued root formation and pulp–dentin regeneration. Cells from distant site, such as systemic circulation, are considered to be cell sources for cell-free REPs. However, these cells make little contribution to pulp regeneration, considering their small numbers.

### Limitations of Cell-Free REPs

**FIGURE 3:**
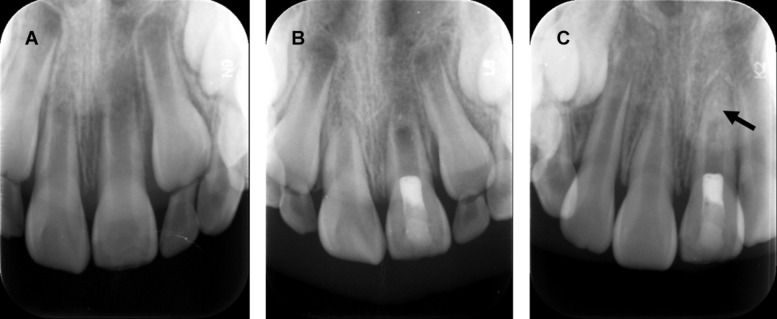
Revascularization promotes continued root development and resolution of periapical lesion, but disorganized radio-opaque changes occur within the apical root canal. **(A)** Radiograph of a maxillary left central incisor: immature root formation with a wide-open apex and periapical lesion. **(B)** Postoperative radiograph of revascularization and coronal restoration. **(C)** Radiograph of one-year follow-up: resolution of periapical lesion and root apex closure. Arrow: non-specific radio-opaque calcific deposit within apical root canal. Courtesy of Dr. Xin Zhou.

Although cell-free REPs are suggested to be effective in eliminating apical periodontitis and even revitalization of non-vital immature teeth in some case reports, their outcomes are still unpredictable. Elimination of apical periodontitis associated with necrotic immature permanent teeth, the primary goal of REPs, can be easily achieved once the infection in the root canal is controlled with disinfection. However, the vitality of cells in apical papilla, dental follicle, and HERS is determined by severity, origin, and duration of inflammation from immature permanent teeth with pulpal necrosis, which is beyond the control of the endodontists. Once severe damage happens to the apical papilla or follicle, there are no dental MSCs supporting odontoblast differentiation or dentin formation, which results in a lack of continued root formation (Figure 3). It is impossible to clearly define the status of MSCs in the apical papilla and dental follicle; thus, endodontists in the clinic always fail to predict the outcomes of cell-free REPs in the necrotic immature permanent teeth.

American Association of Endodontists [AAE] (2016a) has defined REPs as “biologically based procedures designed to physiologically replace damaged tooth structures, including dentine and root structures, as well as cells of the pulp –dentin complex.” This suggests that endogenous stem cells introduced by periapical bleeding might achieve pulp–dentin regeneration, which produce odontoblast-like cells and form dentin. However, both animal and preclinical studies fail to show such results. Formation of bone, cementum, and fibrous tissue is observed with revascularization in dogs. The regeneration of the pulp–dentin complex is rarely detected in the root canal. Additionally, histological studies of human teeth present similar cementum apposition, ectopic bone, and fibrous tissues in human mandibular molars treated with revascularization (Torabinejad and Faras, 2012; Nosrat et al., 2015). Only one human study shows regenerated pulp–dentin complex with odontoblast-like cells and dentin-like tissue in necrotic immature permanent teeth with cell-free REPs, which is assigned to survival of odontoblasts in the root canal (Austah et al., 2018). These studies suggest that the cell-free REPs of necrotic immature permanent teeth are “repair” rather than “regeneration” procedures (Diogenes et al., 2016). Unlike immature teeth with pulpal necrosis, teeth with reversible or irreversible pulpitis can regenerate pulp–dentin complex after cell-free REPs. This might result from the remaining pulp tissue, which means the presence of MSCs responsible for pulp–dentin complex is indispensable for true regeneration in endodontics.

## Cell-Based REPs

### Roles of Dental MSCs in Cell-Based REPs

Mooney et al. (1996) achieved cell-based pulp regeneration by applying pulp cells and polyglycolic acid *in vitro* as early as 1996. In 2005, stem cells were introduced as one of the essential elements of pulp–dentin regeneration in endodontics by Nakashima and Akamine (2005). Soon afterward, Murray et al. (2006) proposed regenerative endodontics as biologically based procedures, in which stem cells play a vital role. Since then, some studies demonstrate the effectiveness of cultured stem cell transplantation in pulp–dentin regeneration (Table 3). Huang et al. (2010) reported that MSC transplantation regenerated pulp–dentin complex in human root fragments compared with formation of fibrous tissue with scaffold alone, which was the first *in vivo* study of pulp–dentin regeneration. Pulp–dentin complex, a layer of odontoblast-like cells on nascent mineralized tissue, is observed in dental MSC–transplanted human dentin with polylactic acid, suggesting a requirement for cell transplantation in pulp–dentin regeneration (Sakai et al., 2010). Later, the necessity of cell transplantation was confirmed with animal studies. Pulp–dentin complex regeneration in large animals was first reported by Iohara et al. (2009) in a pulpotomy model in dogs, in which fractionated side-population cells enriched with CD31^–^/CD146^–^ were transplanted. They also indicate that pulp tissue is regenerated in the root canal with a combination of CD105^+^ DPSCs and SDF-1 (Iohara et al., 2010). SDF-1 is considered an important homing signal by recruiting MSCs to injury sites and facilitating regeneration in various tissues (Suzuki et al., 2011). However, pulp tissue is hardly detected in the root canal with SDF-1 alone. These studies further demonstrate that homing signaling alone is insufficient for pulp–dentin regeneration. The transplantation of pulpal MSCs into the root canal is necessary for pulp–dentin regeneration (Figure 2B).

**TABLE 3:** Current preclinical and clinical studies of REPs based on dental MSCs.

**Study/year**	**Type of dental MSCs**	**Experiment design**						**Results or outcomes**	
		**Animal model**	**Defects**	**Route of administration**	**Biomaterial/scaffold**	**Growth factors**	**Observation period**	**Tissue regeneration**	**Effect evaluation and safety assessment**
**Preclinical studies**									
Iohara et al., 2010	CD 105 + canine DPSCs	60 incisors; 15 dogs	whole pulp removal; enlargement of apical foramen to 0.7 mm	Autologous transplantation; root canal	Mixture of collagen type I & III	Stromal cell-derived factor-1 (SDF-1)	14-90 days	Functional dental pulp	HE: regenerative pulp with well vasculature and innervation on day 14
Iohara et al., 2012	Canine DPSCs	72 incisors; 18 dogs	The whole pulp tissue was removed, and the root canals were enlarged to open the apical foramen to 0.6mmin width in incisors	Autologous transplantation; root canal	Atelocollagen scaffold	Granulocyte-colony stimulating factor (G-CSF)	14-180 days	Functional dental pulp	Safety: no adverse effects on both the whole and local HE: regenerative pulp with well vasculature and innervation on day 14 RG: complete obliteration of the enlarged apical portion and lateral and coronal dentin formation Laser Doppler: functional recovery of pulpal blood flow after 90 days Pulp vitality: positive response on day 60 and day 180
Iohara et al., 2014	canine mobilized DPSCs	16 incisors; 4 dogs	Whole pulp removed, apical foramen enlarged to 0.5 mm	Autologous transplantation; root canal	Atelocollagen scaffold	Granulocyte-colony stimulating factor (G-CSF)	14-120 days	Functional dental pulp	HE: regenerative pulp with well vasculature and innervation on day 14 RG: complete obliteration of the enlarged apical portion and lateral and coronal dentin formation
Nakashima and Iohara, 2014	Canine mobilized DPSCs	-	Root canals after pulpectomy	Autologous transplantation; root canal	Drug-approved collagen	Granulocyte-colony stimulating factor (G-CSF)	14-180 days	Pulp-like tissue	Safety: no adverse effects, no inflammatory cells infiltrated, and no internal or external resorption of the tooth HE: pulplike tissue with well vasculature and innervation was regenerated 14 days RG: complete obliteration of the enlarged apical portion and lateral and coronal dentin formation
El Ashiry et al., 2018	Canine DPSCs	36 incisors; 12 dogs	Pulps from crown and root	Autologous transplantation; root canal	Chitosan hydrogel scaffold	Vascular endothelial growth factor (VEGF-2), basic fibroblast growth factor (bFGF), platelet-derived growth factor (PDGF), nerve growth factor (NGF), bone Morphogenetic protein-7 (BMP7)	1-4 months	Vascularized pulp- dentin like tissue	HE: delicate fibrous tissue resembling the pulp tissue inside the root canal containing multiple large and small blood vessels; newly formed dentin-like tissue with dentinal tubule-like structures along the dentinal walls of the root canal; the regenerated dentin-like tissue did not form well-organized dentinal tubules RGE: closure of the root apex, thickening of the root canal wall, and prolongation of the root lengthening
Xuan et al., 2018	Pig DPSCs	minipigs	Empty root canals after pulpectomy	DPSC aggregates; autologous transplantation; root canals	-	-	3 months	Whole pulp tissue	HE: regenerated pulp tissue containing an odontoblast layer and blood vessels IHC: NeuN

**Study/year**	**Type of dental MSCs**	**Experiment design**						**Results or outcomes**	
		**No. of subjects (teeth)**	**Defects**	**Route of administration**	**Biomaterial/scaffold**	**Growth factors**	**Observation period**	**Tissue regeneration**	**Effect evaluation and safety assessment**

**Clinical studies**									
Nakashima et al., 2017	Human mobilized DPSCs	5 teeth (2 incisors, 3 premolars); 5 patients with irreversible pulpitis	Root canals after pulpectomy	Autologous transplantation; root canal	Atelocollagen scaffold	Granulocyte colony-stimulating factor (G-CSF)	1, 2, 4, 12, and 24/28/32 weeks	Pulp-like tissue	Safety:no adverse events; no postoperative pain, including percussion pain and tenderness; no significant changes in the periapical areas EPT: positive responses after 4 weeks in 4 patients; 1 patient demonstrated a negative response after 24 weeks RG: obliteration of the enlarged apical portion at 24/28 weeks in 3 patients CBCT: lateral dentin formation at 28 weeks in 3 patients MRI: regenerated tissue in the root canal after 24 weeks was similar to that of normal dental pulp in 4 patients
Xuan et al., 2018	Human DPSCs	26 incisors; 36 patients	Dental trauma with pulp necrosis	Two hDPSC Aggregates; Autologous implantation; Root canals	-	Extracellular matrix	1, 3, 6, 9, 12, and 24 months	Whole dental pulp	Safety: no significant side effects after 12 months HE: regeneration of 3D whole dental pulp tissue Digital RVG: no inflammation at the periapical area and continued root development after 24 months EPT: decrease in sensation thresholds CBCT: apical foramen width decreased, the length of the treated tooth root increased Laser Doppler: increase in vascular formation

*HE, hematoxylin and eosin staining; IHC, immunohistochemical staining; RG, radiographic examination; RVG, radiovisiography; EPT, electric pulp vitality testing; CBCT, cone beam computed tomography.*

In recent years, cell-based REPs have aroused growing concern for pulpless teeth. Several clinical studies demonstrate whole dental pulp regeneration. In a preclinical trial, a composite containing human mobilized DPSCs (MDPSCs) and a collagen scaffold was utilized (Nakashima and Iohara, 2014). Upon autologous transplantation with the composite into the root canals of canine mature teeth after pulpectomy, vasculature and innervation- regenerated pulp-like tissue was formed with odontoblast-like cells on the surface of the root dentinal wall and newly formed dentin along the dentinal wall. It suggests that complete dental pulp regeneration similar to healthy dental pulp is achieved along with restoration of tooth function. The rarity of adverse events has confirmed the safety of MDPSC-based REPs. The biological characteristics of MDPSCs do not vary with age, including their stability and regenerative potential. Thus, MDPSCs have been applied to a clinical study to further explore the therapeutic potential and clinical safety of autologous MDPSC transplantation in pulpectomized human teeth (Nakashima et al., 2017). The results show mineralized structure formation of cone beam computed tomography (CBCT), similar signal intensity of magnetic resonance imaging to that of normal dental pulp in untreated controls, robust positive response of a pulp vitality test, and minor adverse events or toxicity. Therefore, human MDPSCs are suggested as safe and efficacious dental MSCs candidates in cell-based REPs.

The therapeutic potential of DPSCs in pulp–dentin regeneration via cell-based REPs is also explored in a minipig pulpectomy model with empty root canals (Xuan et al., 2018). The whole functional pulp tissue regenerates in root canals after implantation of DPSC aggregates harvested from minipigs, consisting of an odontoblast-like layer, blood vessels, and nerves. Based on the preclinical trial with a large animal model, they further conducted a randomized clinical controlled trial to determine the therapeutic effect on immature permanent tooth injuries caused by trauma (Xuan et al., 2018). Those immature necrotic permanent teeth were transplanted autologously with DPSCs collected from primary teeth. Taking apexification as a control group, DPSC-treated immature permanent teeth presented with eliminated apical periodontitis and continued root formation during two years’ follow-up. This was indicated by decreased apical foramen width and increased root length via CBCT, and dentin thickness increased via 3-D reconstruction. The viability of DPSC-treated teeth was validated by laser Doppler flowmetry and electric pulp testing, which showed an increase in vascular formation and decrease in sensation thresholds compared with controls. More excitingly, histological analysis of further traumatized teeth showed regeneration of pulp–dentin complex with an odontoblast layer. Thus, this study demonstrates better efficacy and safety of DPSCs implantation in cell-based REPs, in which 3-D dental pulp tissue with vasculature and innervation was regenerated. Besides, the efficacy and safety of allogenic umbilical cord MSCs have been proved in a preclinical trial (Brizuela et al., 2020). Other cell populations, such as SCAP or non-dental cells might also be useful in cell-based REPs. Considering the accessibility of cell sources, allogeneic cell sources are more usable.

### Challenge for Cell-Based REPs

Cell-based REPs show promising outcomes in pulp–dentin regeneration. Several cell-based REPs are at the stage of clinical studies (Nakashima et al., 2017; Xuan et al., 2018), but transplantation of stem cells is still not recommended by either the American Association of Endodontists [AAE] (2018) or the European Society of Endodontology (ESE) (Galler et al., 2016). Multiple problems needed to be resolved before clinical application of stem cell transplantation, including isolation of stem cells, expansion of cells *in vitro*, practice facilities with good manufacturing, skill of clinicians, training of chair-side assistants, and high cost (Huang et al., 2013). MSCs are one of the most important elements in regenerative endodontics. However, their source and potency are still restrained due to the limitation of our available knowledge. *In vitro*- cultured human somatic stem cells, such as DPSCs, will end up with replicative senescence, a terminal state, after limited cell divisions (Kang et al., 2004). It is suggested that there is a notable elevation of senescent DPSCs cultured *in vitro* and an obvious reduction of odontogenic differentiation potential that may be attributed to loss of stem cell marker, Bmi1 (Mehrazarin et al., 2011). Besides, a large number of cell doublings with homogeneous loss of differentiation potential are required for cell transplantation with *ex vivo* expansion of DPSCs. Due to the aging-related change in DPSCs in the dental tissue of aged patients, the accessibility of DPSCs suitable for regeneration is restricted to immature permanent teeth of young patients. Therefore, lack of DPSCs from pulp tissue would make cell-based REPs in adult permanent teeth difficult to achieve. The *ex vivo* expansion of autologous MSCs in dental appointments with high time restrictions requires practice facilities with good manufacturing, and the procedure is always accompanied by high costs. In this context, allogeneic DPSCs may serve as a potential alternative, which can be produced in high volume and manipulated ready for REPs in the clinic. The immunomodulatory effects of allogeneic MSCs are suggested to be of importance in inflammatory disorders. Allogenic umbilical cord MSCs have been used for mature permanent teeth with combination of plasma-derived biomaterials, showing acceptable safety and efficacy in a phase 1/2 clinical trial (Brizuela et al., 2020). Although transplantation of allogeneic MSCs in REPs shows promising prospects, more research is needed regarding immunogenicity, long-term outcomes, and safety.

## Conclusion

Cell-free REPs, including revascularization and cell homing with molecules recruiting endogenous MSCs, are successful in resolving apical periodontitis and arrested root formation, which are eventually clinical regenerative endodontics and widely applied in treating immature permanent teeth with necrotic pulp. However, histological studies show that pulp–dentin complex is absent in these cases although some studies show a positive response to vitality testing. Instead, cell-based REPs with dental MSCs have shown potential with pulp–dentin regeneration in large animal studies and clinical trials through cell transplantation. Before clinical translation of cell-based REPs, more research is still needed regarding isolation of stem cells, expansion of cells *in vitro*, good practice facilities, skills of clinicians, training of assistants, and reduction of costs. It is hoped that, when cell-based REPs realize true regeneration, they can be applied to the management of necrotic immature permanent teeth, resulting in long-term survival of patients’ natural teeth and dentition.

## Author Contributions

DC and MW conceived the idea and designed the work. SY made the figures. XZ, YL, and YP integrated the materials. LZ revised the manuscript. DC wrote the manuscript. MW revised the manuscript critically. All authors have read and approved the final manuscript.

## Conflict of Interest

The authors declare that the research was conducted in the absence of any commercial or financial relationships that could be construed as a potential conflict of interest.
